# Supplementary figures and images for: Mechanical impact on neural stem cell lineage decisions in human brain organoids
Source: EMBO Rep. 2026 Feb 17;27(6):1393–413. doi: 10.1038/s44319-026-00719-2 (PMC13022303; doi:10.1038/s44319-026-00719-2)

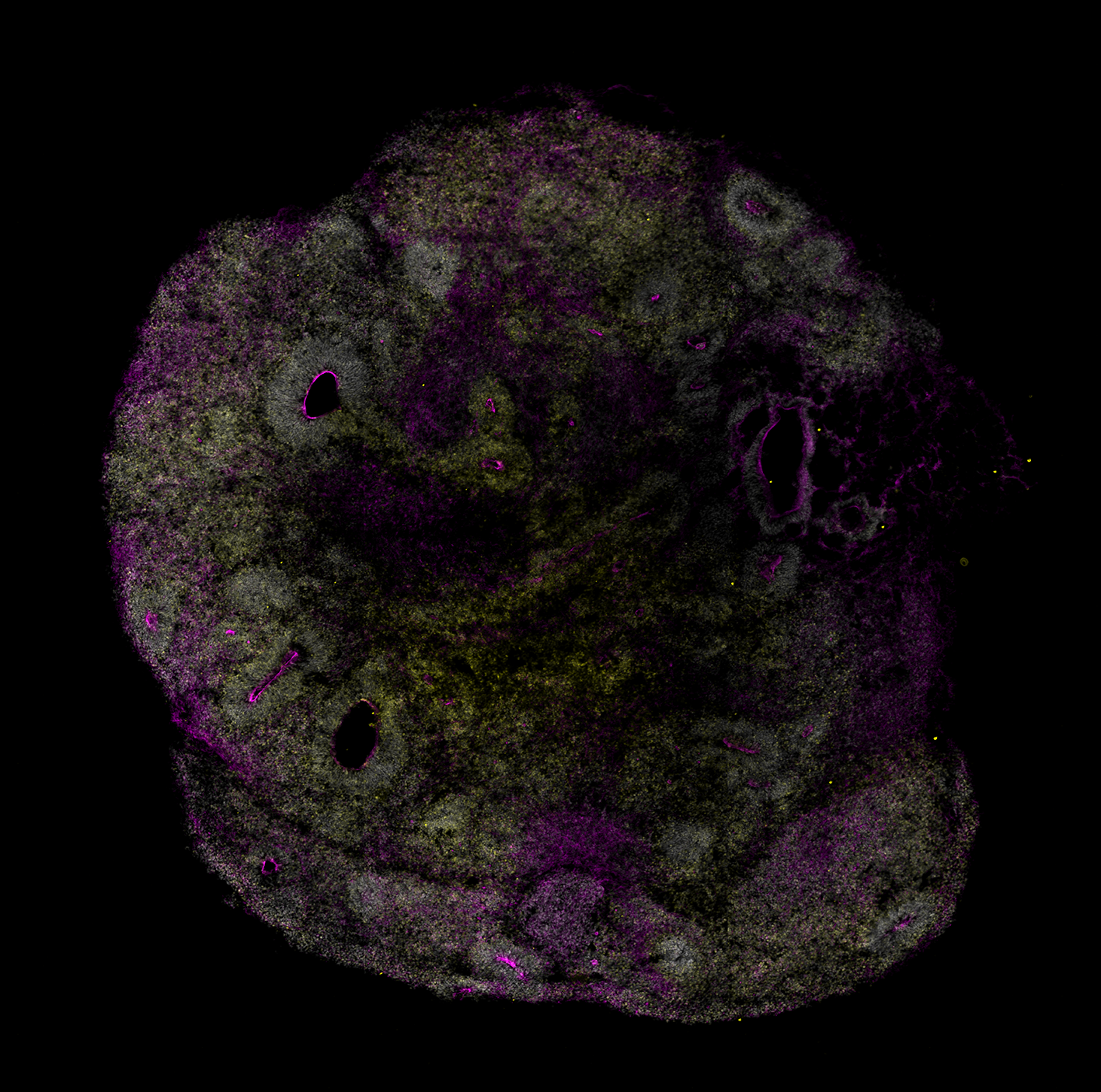

Supplement: Supplementary file 2 — Source data Fig. 1 [file 44319_2026_719_MOESM2_ESM.zip › Figure 1/1B/d30_ctrl_DAPI_SOX2_Phalloidin.tif]

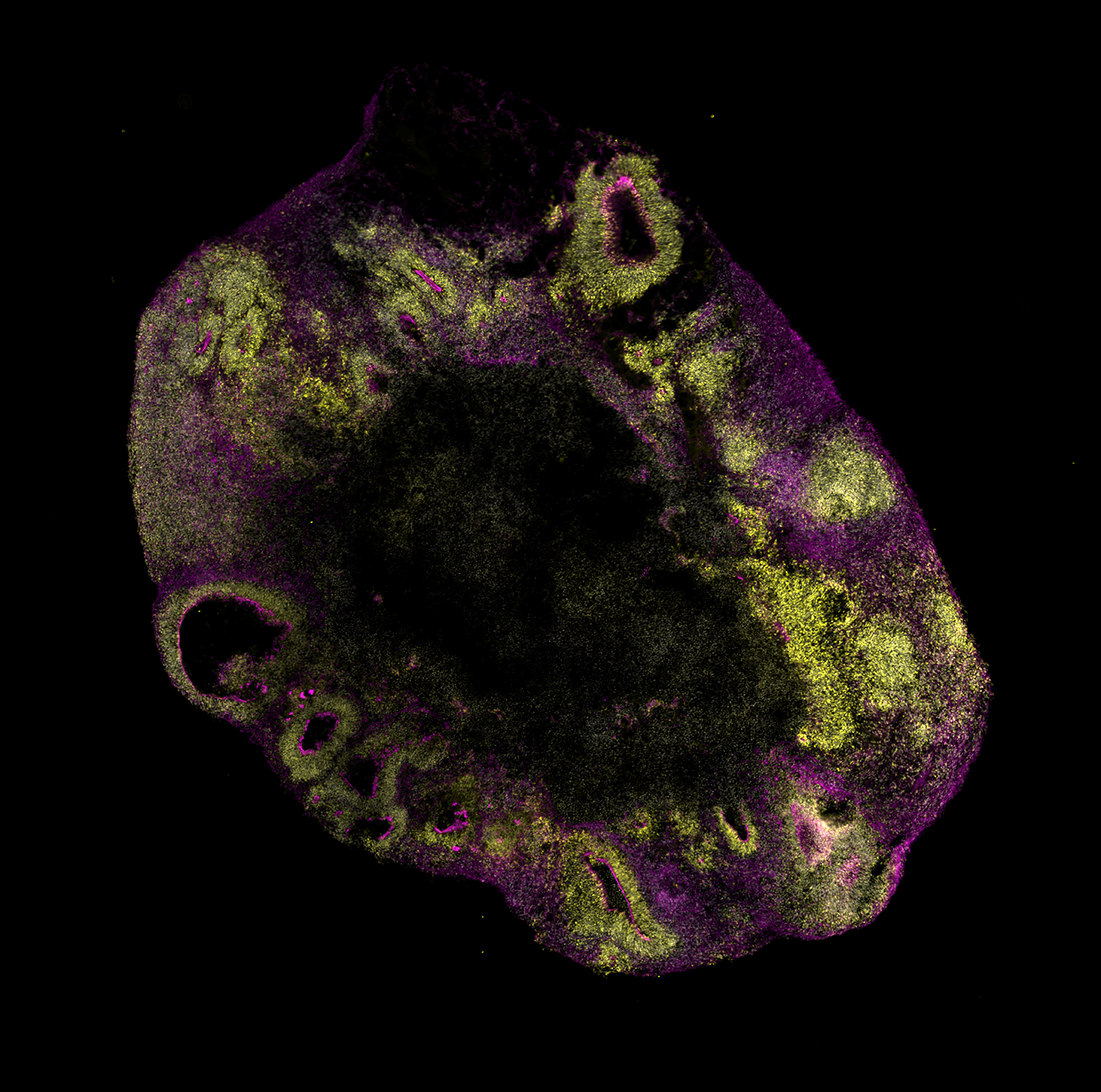

Supplement: Supplementary file 2 — Source data Fig. 1 [file 44319_2026_719_MOESM2_ESM.zip › Figure 1/1B/d30_40%comp_DAPI_SOX2_Phalloifin.tif]

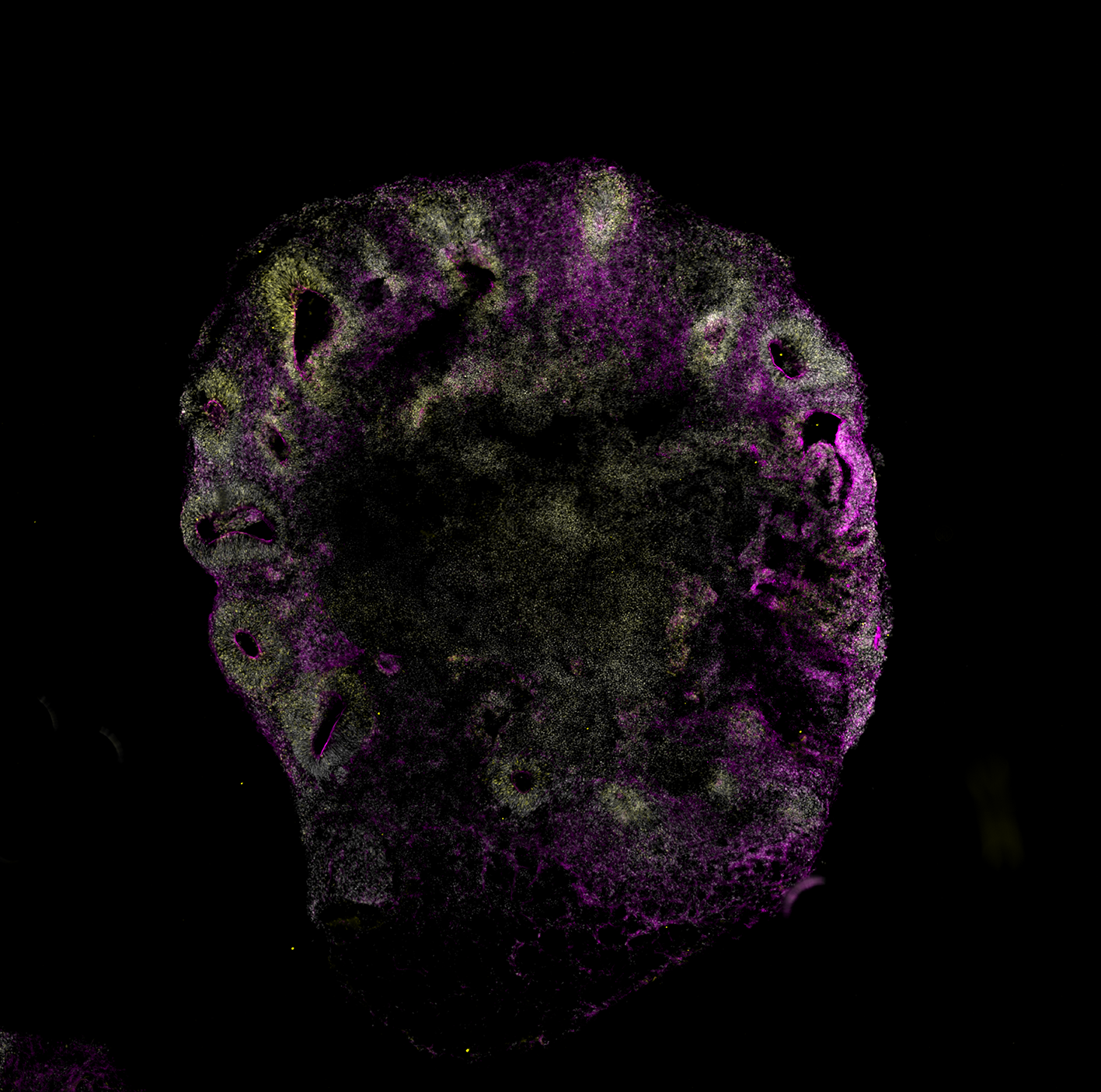

Supplement: Supplementary file 2 — Source data Fig. 1 [file 44319_2026_719_MOESM2_ESM.zip › Figure 1/1B/d30_mock_DAPI_SOX2_Phalloidin.tif]

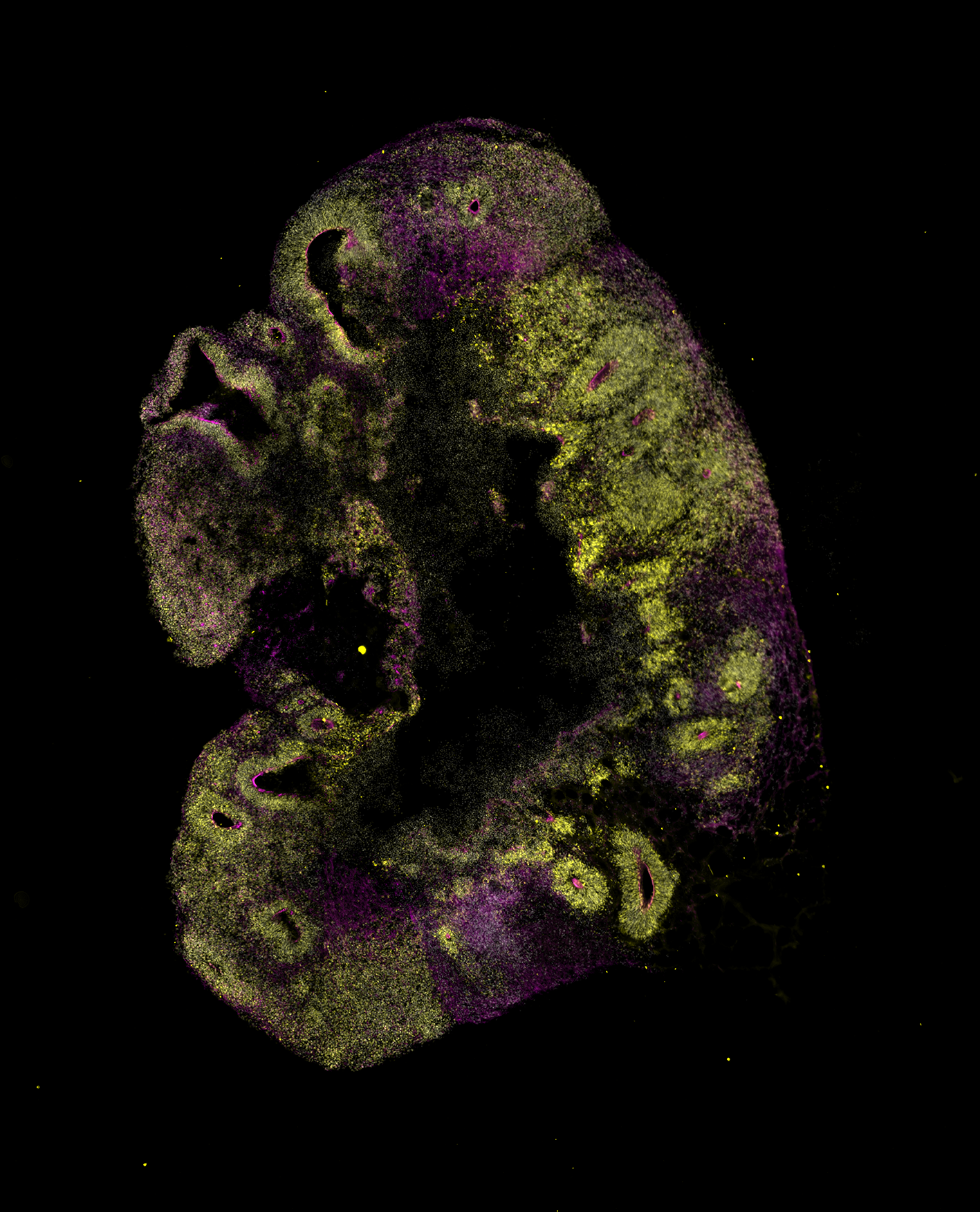

Supplement: Supplementary file 2 — Source data Fig. 1 [file 44319_2026_719_MOESM2_ESM.zip › Figure 1/1B/d30_50%comp_DAPI_SOX2_Phalloidin.tif]

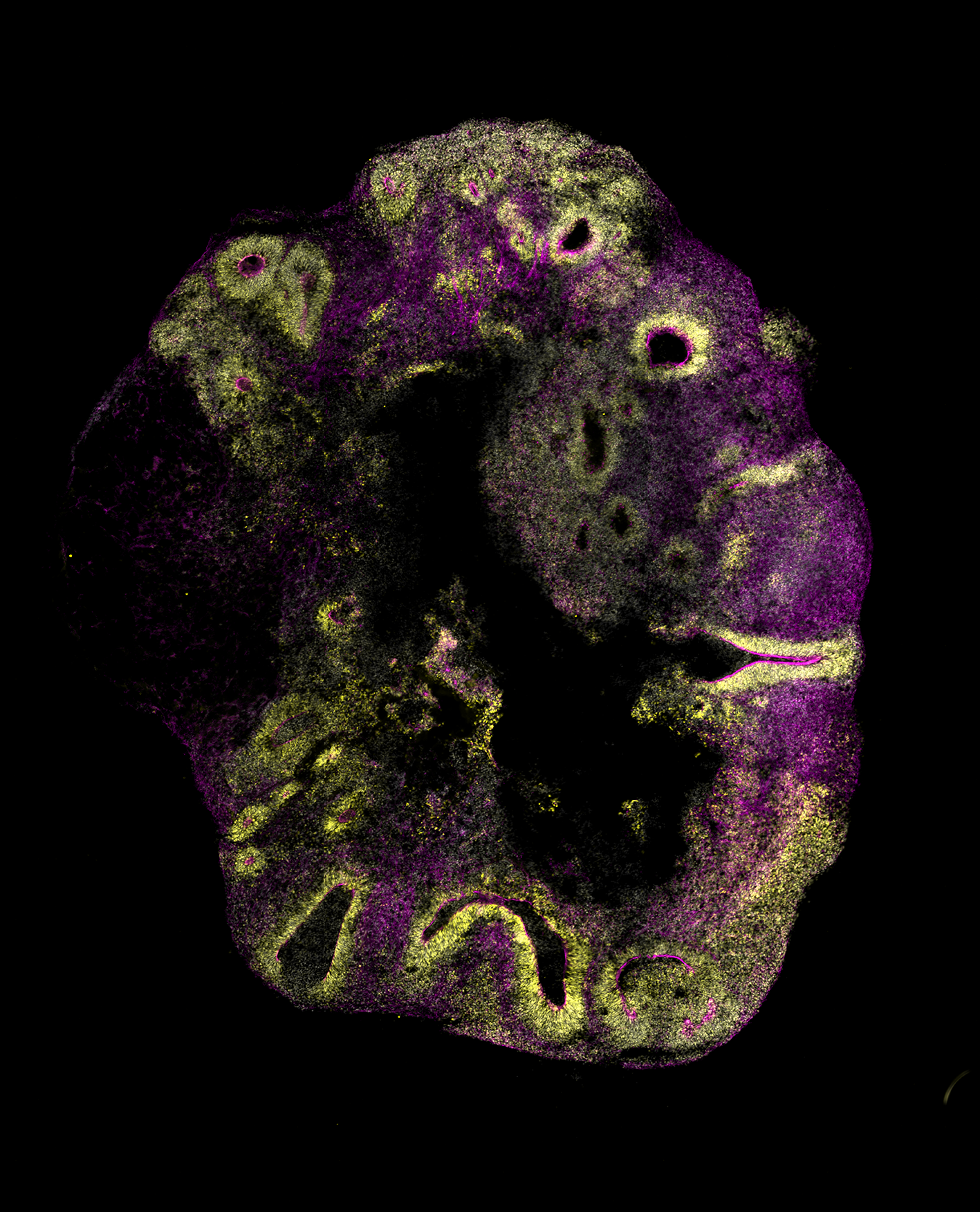

Supplement: Supplementary file 2 — Source data Fig. 1 [file 44319_2026_719_MOESM2_ESM.zip › Figure 1/1B/d30_60%comp_DAPI_SOX2_Phalloidin.tif]

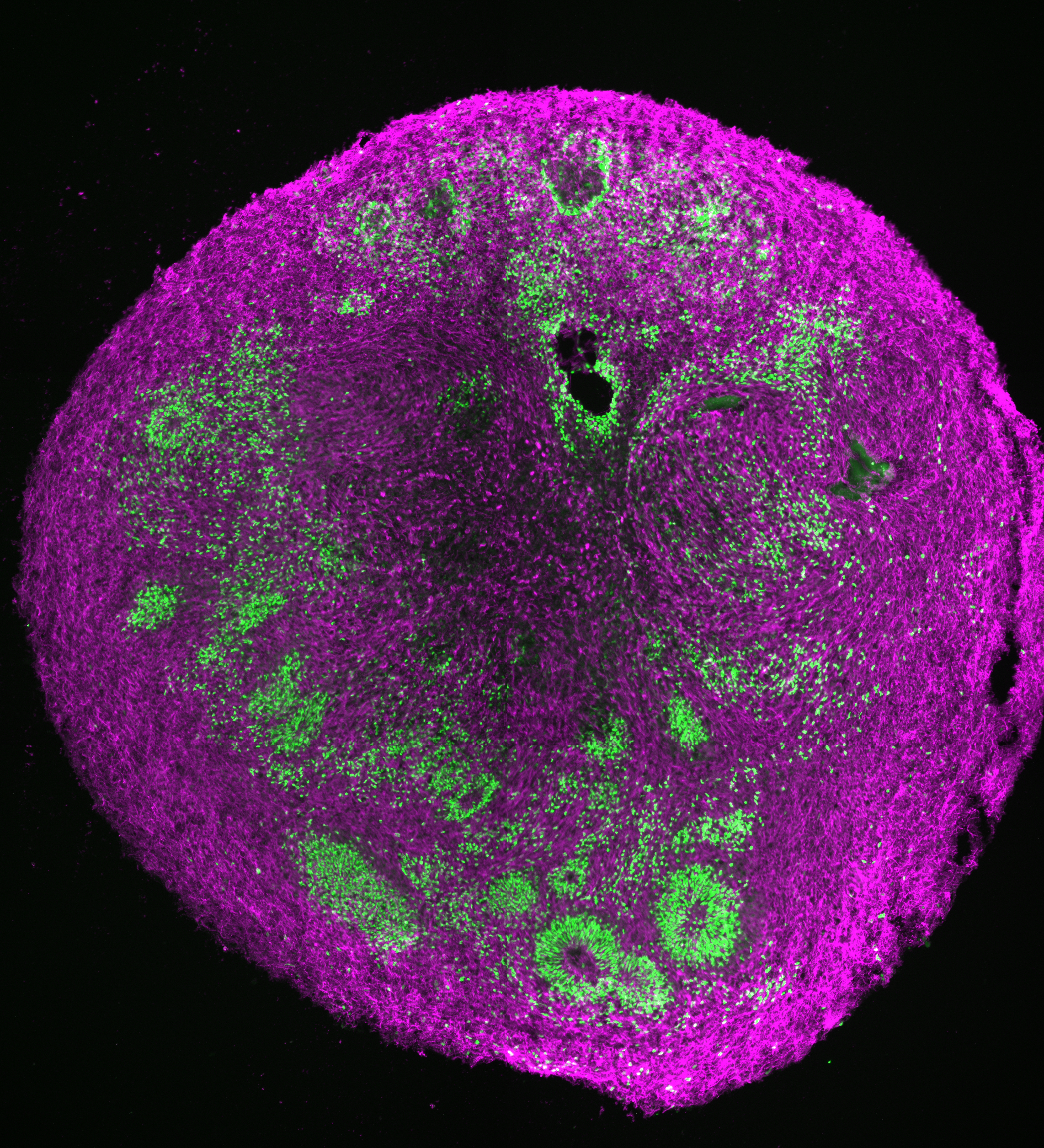

Supplement: Supplementary file 3 — Source data Fig. 2 [file 44319_2026_719_MOESM3_ESM.zip › Figure 2/2B/d70_mock.tif]

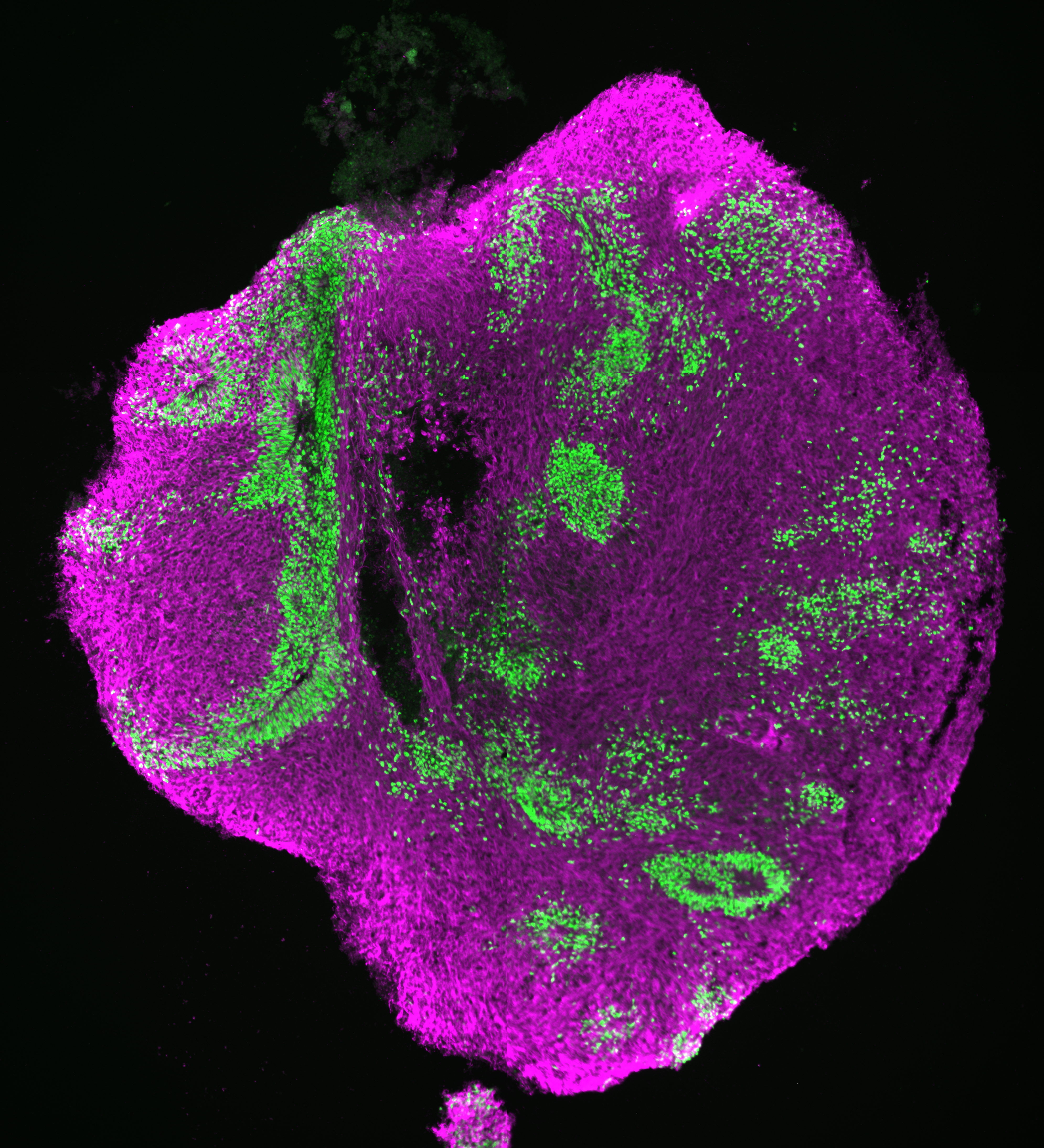

Supplement: Supplementary file 3 — Source data Fig. 2 [file 44319_2026_719_MOESM3_ESM.zip › Figure 2/2B/d70_50%comp.tif]

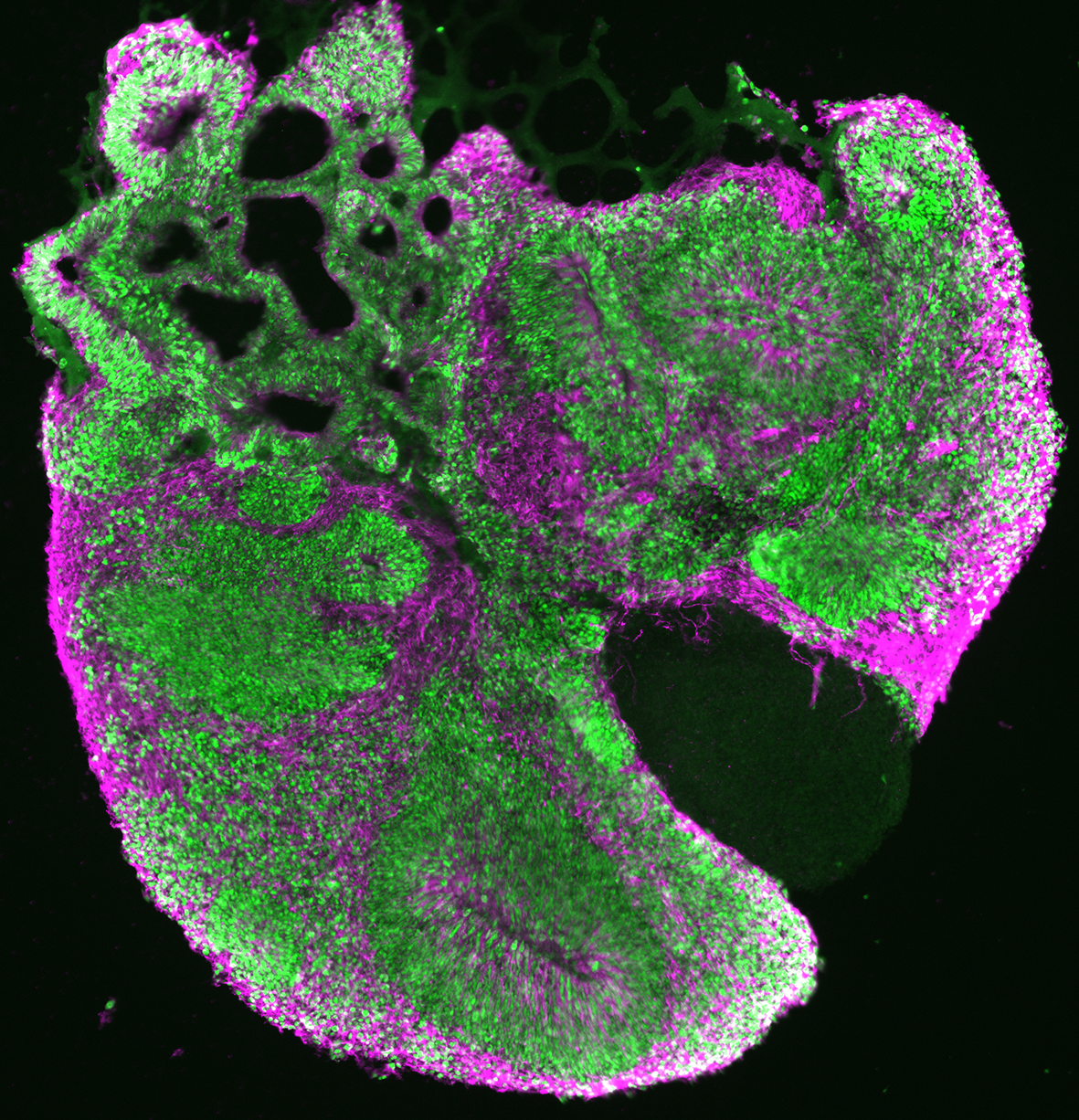

Supplement: Supplementary file 4 — Source data Fig. 3 [file 44319_2026_719_MOESM4_ESM.zip › Figure 3/3G/MG.tif]

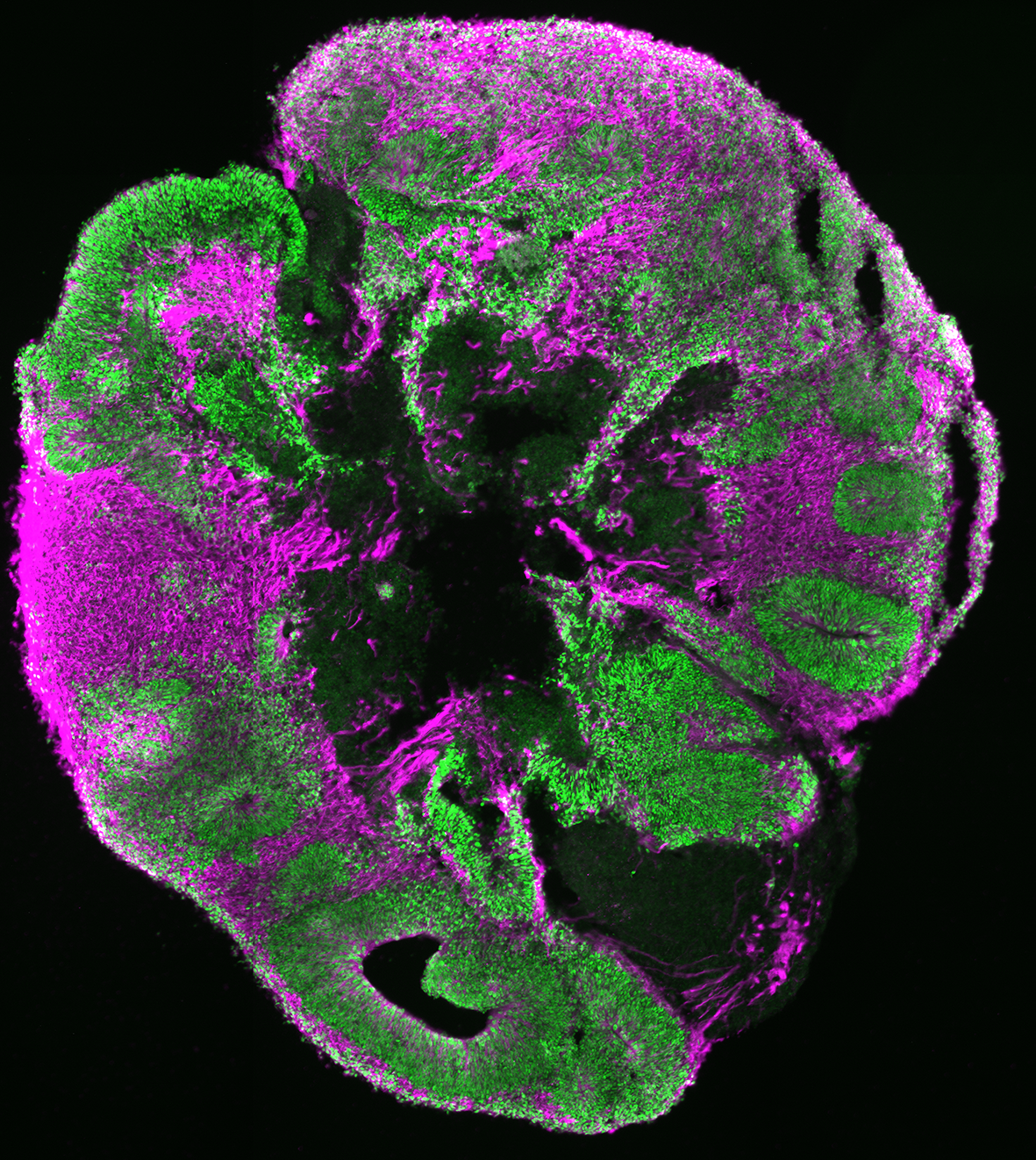

Supplement: Supplementary file 4 — Source data Fig. 3 [file 44319_2026_719_MOESM4_ESM.zip › Figure 3/3G/HG_2,5.tif]

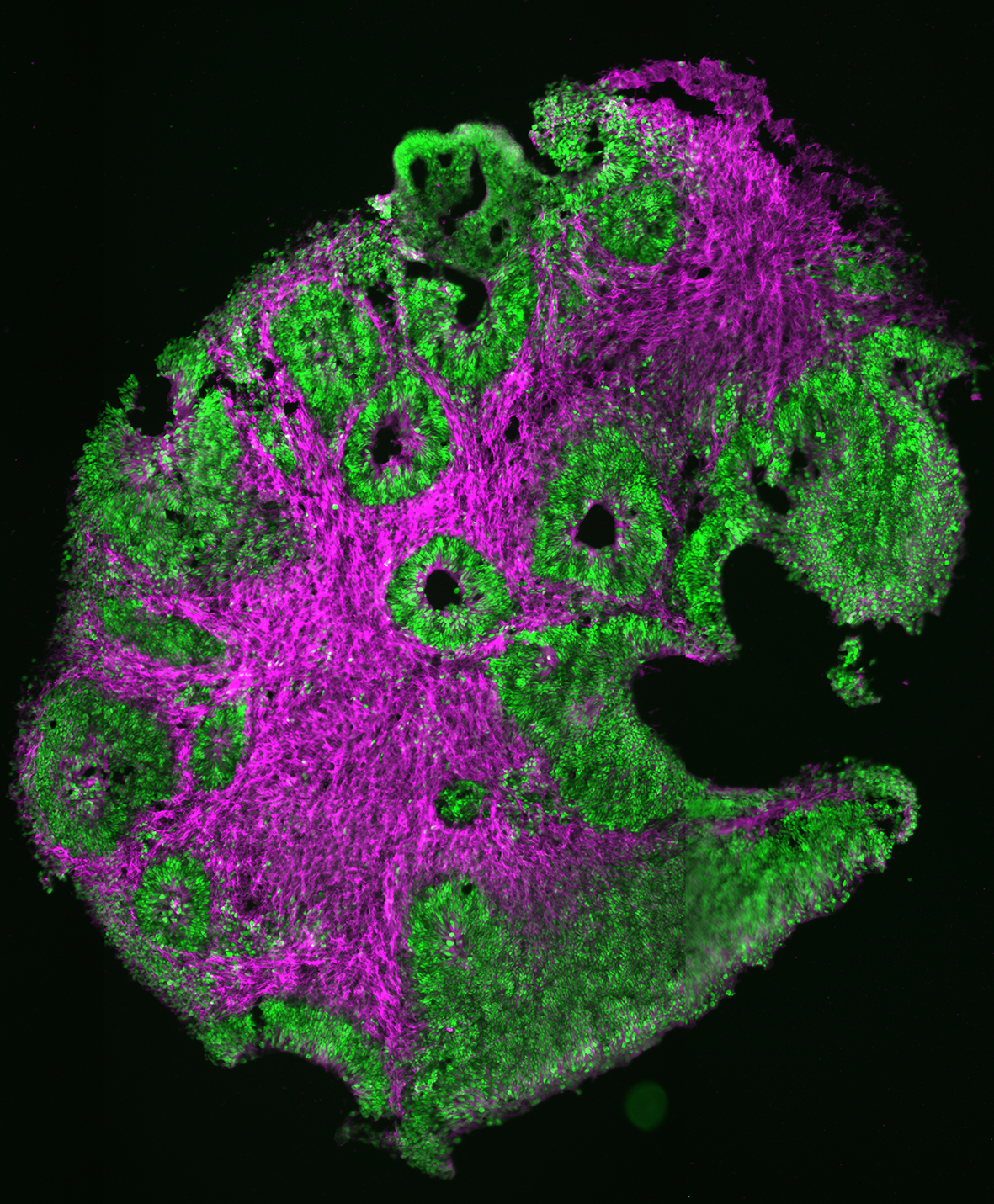

Supplement: Supplementary file 4 — Source data Fig. 3 [file 44319_2026_719_MOESM4_ESM.zip › Figure 3/3G/HG_1,25.tif]
