# Supplementary material for: Mechanical impact on neural stem cell lineage decisions in human brain organoids
Source: EMBO Rep. 2026 Feb 17;27(6):1393–413. doi: 10.1038/s44319-026-00719-2 (PMC13022303; doi:10.1038/s44319-026-00719-2)
Supplement: Supplementary file 5 — Expanded View Figures [file 44319_2026_719_MOESM5_ESM.pdf]

Expanded View Figures

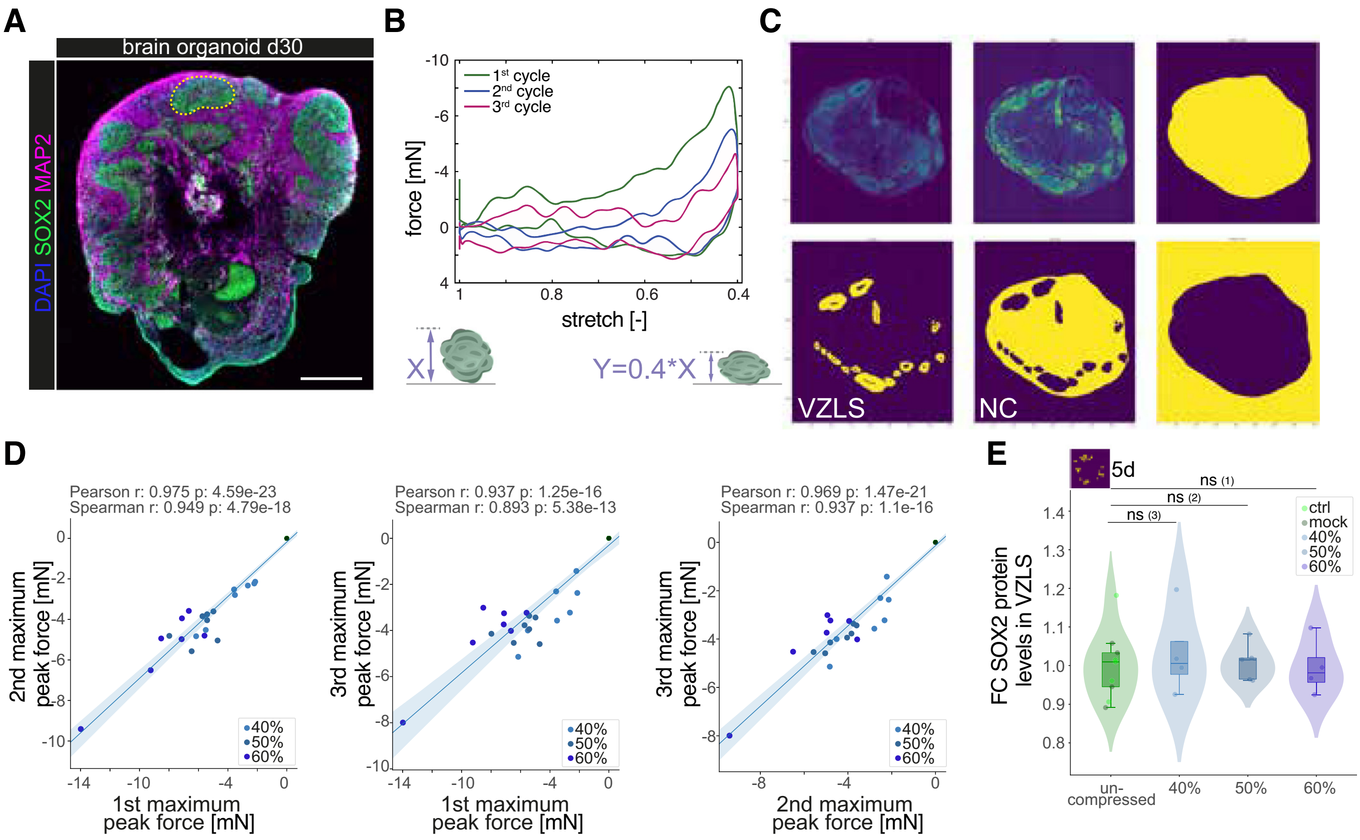

Figure EV1. Acute compression of brain organoids results in transient SOX2 upregulation.

(**A**) Immunofluorescence images of a section from a d30 brain organoid stained for SOX2 (green), MAP2 (magenta), and DAPI (nuclei). Dashed line highlights a VZLS containing SOX2 positive neural stem and progenitor cells, surrounded by MAP2 positive neurons. Scale bar = 500 μm. (**B**) Stretch-force curves measured during cycling compression of a d30 brain organoid. The x-axis shows the degree of deformation (1: no deformation. 0.4: 60% compression). The y-axis shows the applied force. (**C**) Example segmentation of a d30 organoids into ventricular zone-like structures (VZLS) and neuronal compartment (NC) regions. (**D**) Linear regression showing the correlation between the maximum peak force of the first compression cycle and the second cycle (left plot), the first and the third cycle (middle plot), and the second and the third cycle (right plot) across conditions. Pearson as well as Spearman correlations are shown in the plots. Dots represent individual compressed organoids (d30). (**E**) Violin, box, and jitter plots showing the fold change (FC) in mean SOX2 signal in SOX2-positive cells in the VZLS 5 days after compression, relative to uncompressed organoids, based on IF intensity measurements. Note no significant changes in SOX2 levels. Ctrl: $n = 5$; mock: $n = 4$, 40%: $n = 4$; 50%: $n = 5$; 60%: $n = 4$; Exact P values: (1): 1.000; (2): 5.185×10^{-1}; (3): 7.105×10^{-1}. Violin plots show the distribution of the data. Boxplots show the median (center line), interquartile range (box), and whiskers extending to the most extreme values within 1.5× the interquartile range; dots represent individual organoids. Statistical significance was assessed using a two-sided Wilcoxon rank-sum test (ns: $P > 0.05$; *: $P < 0.05$; **: $P < 0.01$; ***: $P < 0.001$). Yellow/magenta images in the upper left corner indicate whether VZLS and/or NC were included (according to the segmentation shown in Fig. EV1C).

 © The Author(s)

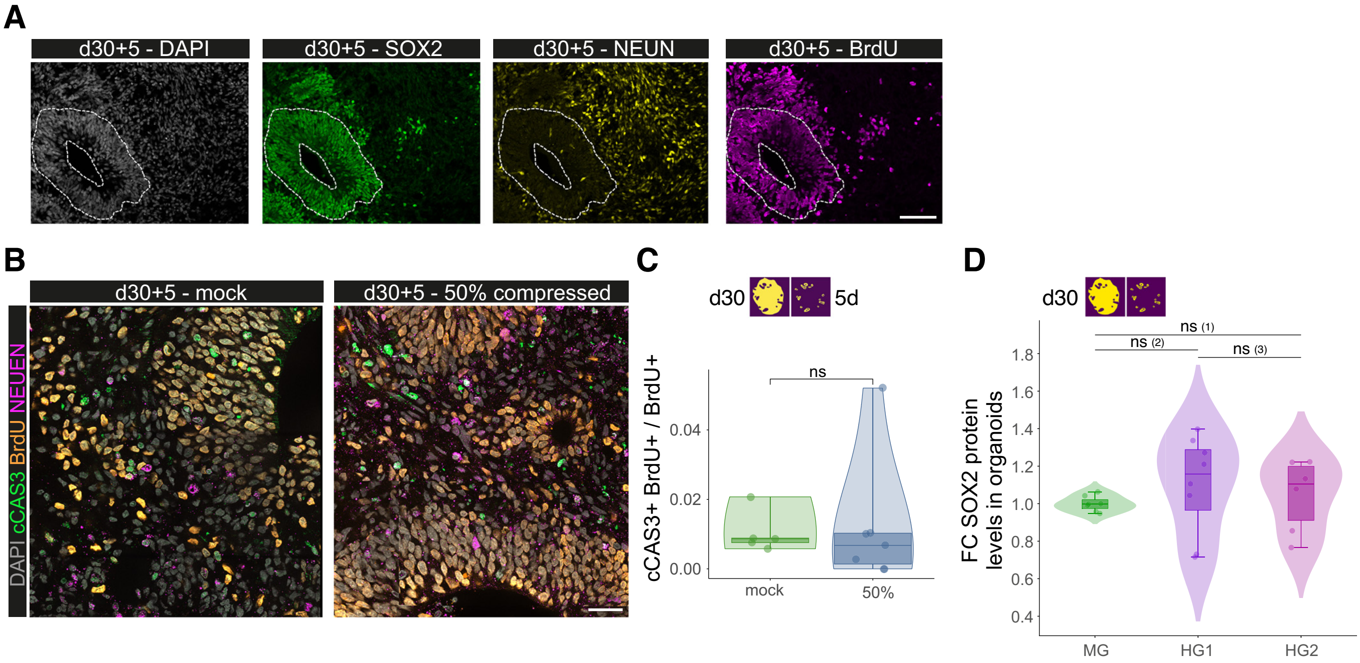

Figure EV2. Long-lasting changes on molecular and cell fate level.

Immunofluorescence images of section from d30 organoids 5 days after compression and BrdU addition showing co-staining of the stem cell marker SOX2, the neuronal marker NEUN and the incorporation of BrdU (experimental paradigm: Fig. 4B). Dashed line delineates a VZLS. Scale bar = 100 µm. (**B**) Immunofluorescence images showing BrdU, cCAS3, NEUN co-staining in mock control (left image) and 50% compressed d30 organoids (right image) 5 days after compression and BrdU addition. DAPI was used to counterstain for nuclei. Scale bar = 50 µm. (**C**) Violin, box, and jitter plots showing the fraction of cleaved caspase 3 (cCAS3)/BrdU double positive cells among all BrdU positive cells to reveal the fraction of apoptotic cells among the cells born after compression. Mock: $n = 5$, 50%: $n = 7$; exact P value: 0.63. (**D**) Violin, box, and jitter plots showing the fold change (FC) in mean SOX2 signal in SOX2-positive cells in whole organoid sections of d30 organoids embedded in different matrixes, relative to MG embedded organoids, based on IF intensity measurements. MG = matrigel; HG1 = 1.25% OHA/2.5% GEL; HG2 = 2.5% OHA/2.5% GEL; dots represent individual organoids. MG: $n = 6$; HG1: $n = 8$; HG2: $n = 6$. Exact P values: (1): 1.520×10^{-1}; (2): 3.660×10^{-1}; (3): 7.546×10^{-1}. In (**C**, **D**), Violin plots show the distribution of the data. Boxplots show the median (center line), interquartile range (box), and whiskers extending to the most extreme values within 1.5× the interquartile range; dots represent individual organoids. Statistical significance was assessed using a two-sided Wilcoxon rank-sum test (ns: $P > 0.05$). Yellow/magenta images in the upper left corner indicate whether VZLS and/or NC were included (according to the segmentation shown in Fig. EV1C).

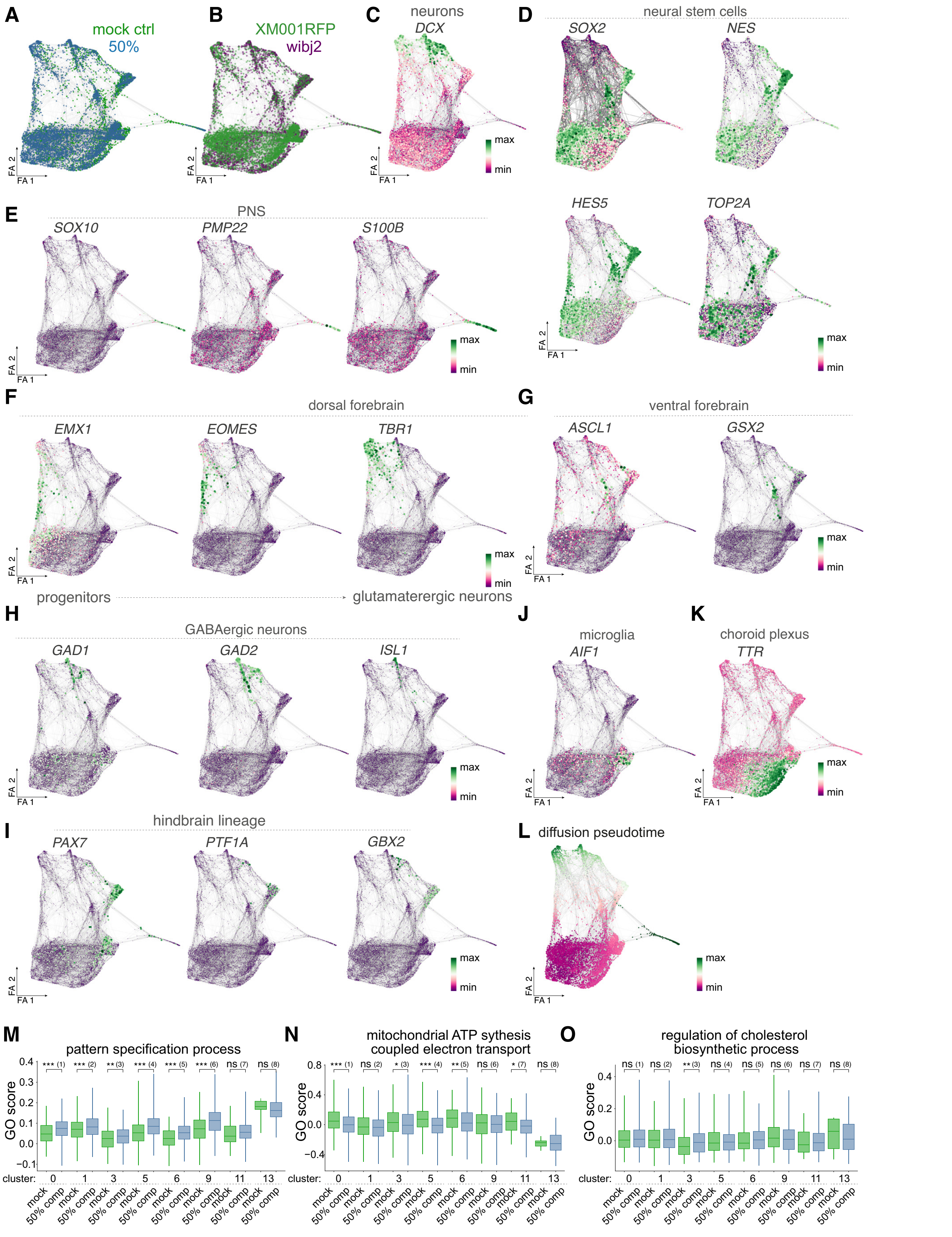
A
mock ctrl
50%
B
XM001RFP
wibj2
C
neurons
DCX
D
neural stem cells
SOX2
NES
HES5
TOP2A
E
PNS
SOX10
PMP22
S100B
F
dorsal forebrain
EMX1
EOMES
TBR1
G
ventral forebrain
ASCL1
GSX2
progenitors
glutamaterergic neurons
H
GABAergic neurons
GAD1
GAD2
ISL1
J
microglia
AIF1
K
choroid plexus
TTR
I
hindbrain lineage
PAX7
PTF1A
GBX2
L
diffusion pseudotime
max
min
FA 1
FA 2
M
pattern specification process
N
mitochondrial ATP sythesis coupled electron transport
O
regulation of cholesterol biosynthetic process
GO score
cluster: 0 1 3 5 6 9 11 13
mock
50% comp
*** (1) *** (2) ** (3) *** (4) *** (5) *** (6) ns (7) ns (8)
*** (1) ns (2) * (3) *** (4) ** (5) ns (6) * (7) ns (8)
ns (1) ns (2) ** (3) ns (4) ns (5) ns (6) ns (7) ns (8)

 © The Author(s)

Figure EV3. Molecular dissection of the impact of compression on single-cell level.

(**A**) force-directed graph embedding showing the contribution of mock and compressed cells, as well as (**B**) of the two different hiPSC lines XM001RFP and wibj2 used. Force-directed graph embedding showing (**C**) expression of the neuronal marker *DCX*, and (**D**) expression of the neural stem cell marker genes *SOX2, NES, HES5* and the proliferation marker *TOP2A*. (**E**) Expression of PNS markers (*SOX10*, *PMP22*, and *S100B*). (**F**) Expression of dorsal forebrain trajectory markers with the neural stem and progenitor marker *EMX1*, *EOMES* marking intermediate progenitor and the neuronal marker *TBR1*. (**G**) Expression of markers for ventral forebrain lineage cells (*ASCL1, GSX2*) and (**H**) GABAergic neurons (*GAD1, GAD2, ISL1*). (**I**) Expression of hindbrain lineage markers (*PAX7, PTF1A, GBX2*). (**J**) *AIF1* expression highlighting the microglia cluster. (**K**) Expression of choroid plexus marker (*TTR*). (**L**) Diffusion pseudotime plotted on the force-directed graph embedding. (**A–I**) FA refers to force atlas. The size of the dots indicates the extent of expression. (**M**) Boxplot showing the expression score of the GO term 'pattern specification process' within specific clusters comparing mock and 50% compressed samples, respectively. Exact P values: (1): 4.8×10^{-10}; (2): 2.0×10^{-4}; (3): 6.5×10^{-3}; (4): 6.8×10^{-11}; (5): 2.5×10^{-6}; (6): 4.2×10^{-8}; (7): 0.086; (8): 0.69. (**N**) Boxplot showing the expression score of the GO term 'mitochondrial ATP synthesis coupled electron transport' within specific clusters comparing mock and 50% compressed samples, respectively. Exact P values: (1): 3.4×10^{-6}; (2): 0.076; (3): 0.045; (4): 4.6×10^{-9}; (5): 9.9×10^{-3}; (6): 0.87; (7): 0.022; (8): 1. (**O**) Boxplot showing the expression score of the GO term 'regulation of cholesterol biosynthetic process' within specific clusters comparing mock and 50% compressed samples, respectively. Exact P values: (1): 0.41; (2): 0.87; (3): 2.1×10^{-3}; (4): 0.78; (5): 0.22; (6): 0.63; (7): 0.84; (8): 0.9. For (**M**, **N**, **O**), Boxplots show the median (center line), interquartile range (box), and whiskers extending to the most extreme values within 1.5× the interquartile range; dots represent individual organoids. Statistical significance was assessed using a two-sided Wilcoxon rank-sum test (ns: $P > 0.05$; *: $P < 0.05$; **: $P < 0.01$; ***: $P < 0.001$). For this analysis all neural stem and progenitor cells as well as the more differentiated neurons are included (cluster 0, 1, 3, 5, 6, 9, 11, 13 in Fig. 5A). Number of cells in each cluster (mock/50% compressed): (0): 220/1175; (1): 324/819; (3): 229/390; (5): 295/312; (6): 125/427; (9): 168/220; (11): 53/173; (13): 5/97.

© The Author(s)
